# Essential Role of Non-Coding RNAs in Enterovirus Infection: From Basic Mechanisms to Clinical Prospects

**DOI:** 10.3390/ijms22062904

**Published:** 2021-03-12

**Authors:** Peiyu Zhu, Shuaiyin Chen, Weiguo Zhang, Guangcai Duan, Yuefei Jin

**Affiliations:** 1 Department of Epidemiology, College of Public Health, Zhengzhou University, Zhengzhou 450001, China; zpy6860@gs.zzu.edu.cn (P.Z.); sychen@zzu.edu.cn (S.C.); wzhang033@icloud.com (W.Z.); gcduan@zzu.edu.cn (G.D.); 2 Department of Immunology, Duke University Medical Center, Durham, NC 27710, USA

**Keywords:** non-coding RNAs, enteroviruses, immune dysfunction, apoptosis, signaling pathway

## Abstract

Enteroviruses (EVs) are common RNA viruses that can cause various types of human diseases and conditions such as hand, foot, and mouth disease (HFMD), myocarditis, meningitis, sepsis, and respiratory disorders. Although EV infections in most patients are generally mild and self-limiting, a small number of young children can develop serious complications such as encephalitis, acute flaccid paralysis, myocarditis, and cardiorespiratory failure, resulting in fatalities. Established evidence has suggested that certain non-coding RNAs (ncRNAs) such as microRNAs (miRNAs), long ncRNAs (lncRNAs), and circular RNAs (circRNAs) are involved in the occurrence and progression of many human diseases. Recently, the involvement of ncRNAs in the course of EV infection has been reported. Herein, the authors focus on recent advances in the understanding of ncRNAs in EV infection from basic viral pathogenesis to clinical prospects, providing a reference basis and new ideas for disease prevention and research directions.

## 1. Introduction

Enteroviruses (EVs) are a genus of small, single-stranded, positive-sense RNA viruses of the family *Picornaviridae* [1]. These diverse EVs are associated with a variety of human diseases including hand, foot, and mouth disease (HFMD), poliomyelitis, viral myocarditis, neonatal sepsis-like disease, encephalitis, acute flaccid paralysis (AFP), and respiratory diseases [2]. EVs consist of 13 species, seven of which are human pathogens. Including three serotypes of poliovirus, these seven EV species contain approximately 300 subtypes [2] and include coxsackieviruses, echoviruses, and numbered Enterovirus A to D (Table 1) [3]. In the past few decades, more attention has been paid to poliovirus, but in recent years, outbreaks of non-polio EVs have emerged as serious public health concerns [2]. These non-polio EVs include EVA71, which has caused epidemics of HFMD in the Asia–Pacific region [1], EV-D68, which is responsible for epidemics of severe lower respiratory tract disease in North America [4], and coxsackieviruses (CV), which are a major cause of viral myocarditis (Table 1) [5]. Nonetheless, specific antiviral drugs for non-polio EV infections are not currently available, and the proven benefits of several vaccines remain unclear (e.g., EVA71 vaccines). To overcome these limitations in the control of EV infection, a comprehensive understanding of virus–host interactions and viral pathogenesis is urgently needed.

## 2. The Epidemiological Characteristics of EVs

EV infection can lead to a broad disease spectrum, from minor febrile illness and rashes to severe and sometimes fatal conditions including meningitis, encephalitis, paralysis, and myocarditis [3]. EVs are mainly transmitted by the fecal–oral route or via pathogen-laden respiratory droplets [3]. EV infections typically exhibit seasonal patterns of incidence in both temperate and tropical climates, although seasonality is usually more obvious in the former, with infections more common in the summer and early fall [3]. As a common infectious disease, infants and children seem more susceptible to infection. In general, the majority of infections are subclinical, but sporadic or regular outbreaks of EVs-associated disease are often worldwide and can lead to significant morbidity and mortality [3]. In particularly, non-polio EVs have been recognized as an emerging cause of neurological diseases [6]. This issue has become more serious in recent years. For example, the HFMD outbreaks have increased in Europe and in the Asia–Pacific region (Cambodia, Japan, Malaysia, Singapore, South Korea, China, Thailand, and Vietnam) [1,3]. Despite the elimination of polio in most countries, the burden of AFP remains high, with many of those infections associated with non-polio EVs. Severe lower respiratory tract infections caused by EV-D68 also pose a great threat to human health in North America, Italy, Japan, the Netherlands, and the Philippines [7,8,9,10,11].

EV particles share a similar icosahedral structure, which is constructed of 60 repeating protomers, with each protomer consisting of four structural proteins VP1–VP4. Among them, VP4 is myristoylated and located on the inside surface of the virion capsid, while the outer surface of the particle is composed of subunits of VP1, VP2, and VP3 [2]. Most EVs possess a deep, spherical surface concavity or canyon-encircling each fivefold axis of symmetry that frequently acts as the receptor binding site [2]. The EV lifecycle begins with binding to cell surface receptors, leading to receptor-mediated endocytosis. After delivery to the cytosol, the viral RNA is translated into a single large polyprotein (VPg). The VPg is further cleaved by viral proteinases 2A, 3C, and 3CD into ten proteins including structural capsid proteins (VP0, VP1, and VP3), replication proteins (2A–2 C and 3A–3D), and some stable and functional cleavage intermediates [2]. EV particles form by assembly of VP0, VP1, and VP3 into protomers and pentamers. Together with a primeval viral RNA, pentamers form the provirion. Finally, the RNA-promoted procedure transforms VP0 into VP2 and VP4 to produce mature virions [2].

## 3. Discovery and Concept of Non-Coding RNAs (ncRNAs)

Due to advances in RNA sequencing techniques, thousands of non-coding transcripts with an unknown function have been discovered [12], which are broadly referred to as non-coding RNA (ncRNAs). There are three main types of ncRNAs, namely microRNAs (miRNA), long non-coding RNAs (lncRNA), and circular RNAs (circRNA) that consist of a closed continuous loop [12]. Accumulated evidence suggests that ncRNAs serve as regulators of almost every cellular process, and their expression appears to be strictly modulated in physiological conditions and in some human diseases, including infectious diseases [13,14]. The emerging links between ncRNAs and human diseases provide a new direction for the development of therapeutic and diagnostic approaches [13]. This review focuses on and summarizes current knowledge on the critical roles of ncRNAs in EV infection and provides perspectives on the development of antiviral strategies mediated by these important regulatory molecules.

## 4. Description and Biogenesis of ncRNAs

NcRNAs are important components of the transcriptome and play a critical role in both normal physiology and pathological processes such as infection and carcinogenesis [12,13,15]. The biosynthesis of these transcripts occurs in the nucleus much like any protein-coding RNAs, but ncRNAs lack an open reading frame (ORF) and thus cannot be transcribed into proteins [12]. NcRNAs are divided into lncRNAs and smaller ncRNA species such as miRNAs and others according to the size of their nucleotides. The term lncRNA generally refers to transcripts with more than 200 nucleotides and do not possess protein-coding functions [13]. MiRNAs are defined as single-stranded ncRNAs of ≈20 nucleotides in length, which are processed by Dicer from a hairpin precursor [13]. MiRNAs were first described in the worm *Caenorhabditis elegans* [16], and since then, numerous of miRNAs have been recognized both in plants and mammals [17,18]. LncRNAs regulate gene expression in the nucleus through the interaction with DNA, chromatin-modifying complexes, and/or various transcriptional regulators. In addition, cytoplasmatic lncRNAs can serve as “sponges” for other transcripts or proteins by acting as protein templates themselves or by regulating the degradation and translation efficiency of mRNAs [12]. Unlike lncRNAs, miRNAs regulate gene expression at the post-transcriptional level by binding to target sites within mRNAs and viral genomes [13,15]. Naturally occurring miRNA-targeting sites within viral genomes are commonly located in the 5′ and 3′ non-translated regions (NTRs), but they have recently been detected in the coding regions of viral proteins [13]. Increasing evidence indicates that the expression of ncRNAs is not limited to classical mechanisms. CircRNAs represent another group of ncRNAs that form a single-stranded closed loop structure with covalent bonds [13]. Some circRNAs are highly abundant in eukaryotes, are evolutionarily conserved, can be highly specific for certain cell types or developmental stages, and are extraordinarily stable [13].

LncRNAs are produced and processed in the nucleus similar to protein coding transcripts [13]. As with mRNAs, most lncRNAs are transcribed by RNA polymerase II (Pol II) and exist in different parts of the cell [13]. Several lncRNAs are known to be released into the cytoplasm after their biogenesis and processing, while the majority remain in the nucleus and are recruited to the chromatin [13]. The synthesis of miRNAs begins with the transcription of a primary transcript by Pol II, from which a primary miRNA (pri-miRNA) is produced. The pri-miRNA is further cleaved by Drosha, a nuclease of the RNase III family, leading to a pre-miRNA [15,19]. The pre-miRNA is subsequently exported from the nucleus into the cytoplasm, which is a process mediated by Exportin-5 (XPO5), and it is then enzymatically cleaved by Dicer into the miRNA duplex [15,19]. One strand of the miRNA duplex is recognized by the Ago proteins to form the RNA-induced silencing complex (RISC), which regulates the expression of target genes by degradation or translational suppression [15,19,20]. By contrast, circRNAs are generated from splicing events and subsequent exon or intron circularization events [21]. Exonic circRNAs depend on spliceosomal action and can be produced by a process that is referred to as a backsplicing variant of expression [13].

## 5. Acute EV Infection and ncRNA Expression Profiles

Several studies have investigated the impact of acute EV infection on the expression profiles of cellular miRNAs and lncRNAs using RNA-seq and microarray (Table 2). Most of these studies focused on either EVA71 and HFMD, or CVB3 and viral myocarditis (see table for references). Altered circulating miRNA and lncRNA profiles have also been described in EV infection. However, due to different experimental conditions (cell lines or tissue, time points post-infection, host factors, virus strains used, and other factors), there was little overlap in the miRNAs identified as dysregulated by these different studies. Thus, much work remains in future studies to help clarify and validate these processes in a manner that can be more broadly applied to various EV subtypes during acute infection.

## 6. Known Impacts of ncRNAs on EV Replication

Evidence increasingly suggests that diverse ncRNAs play vital roles in viral infection, either for the benefit of the host cell or for the virus itself [12]. Regarding the latter scenario, viruses can hijack various ncRNAs to modulate the expression of both cellular and viral genes to establish an environment conducive to its own replication [38]. By contrast, certain dysregulated ncRNAs can also directly or indirectly affect viral replication and even directly target viral genomes [38,39]. Some reported observations of these phenomena, particularly involving the activity of miRNAs, are as follows.

Ectopic miR-10a*, which is the star strand of the RNA duplex, induced CVB3 biosynthesis by targeting the 3D-coding region [40], while ectopic miR-342-5p suppressed CVB3 replication by targeting the 2C-coding sequence [41]. The increase in miR-203 [42] and miR-20b [43] benefit CVB3 replication by suppressing zinc finger protein-148 (ZFP-148), while miR-126 promoted the replication of CVB3 by influencing several cellular signaling pathways [44]. The miR-221/222 cluster was significantly induced during acute viral myocarditis caused by CVB3, which further decreased cardiac viral load by targeting E-twenty six 1/2 (ETS1/2), Interferon regulatory Factor 2 (IRF2), Bcl-2-like protein 11 (BCL2L11), TOX, Bcl-2-modifying factor (BMF), and CXC chemokine ligand 12 (CXCL12) [45]. By contrast, miR-296-5p decreased EVA71 replication by interacting with nt 2115 to 2135 and nt 2896 to 2920 of the viral genome [46]. The biosynthesis of EVA71 in host cells also could be inhibited by miR-373 and miR-542-5p, which directly targeted the 5′-UTR of the viral genome [47]. The overexpression of miRNA-2911 encoded by *Honeysuckle* reduced EVA71 replication by directly targeting the VP1-coding sequence [48]. Loss of function of scavenger receptor class B member (SCARB2), a receptor for EVA71 entry, was caused by miR-127-5p, which led to a decrease in viral replication [49]. Most dysregulated miRNAs during EVA71 infection can interact with host signaling pathways, indirectly affecting virus replication [50,51,52]. For example, the downregulation of miR-27a with the target of epidermal growth factor receptor (EGFR) increased EVA71 biosynthesis [50]. In addition, EVA71-induced hsa-let-7c-5p could promote viral replication by mitogen-activated protein kinase kinase kinase kinase-4 (MAP4K4) degradation [51]. Furthermore, miR-30a binding to the 3′-UTR of Beclin-1 transcripts decreased the expression of Beclin-1, resulting in reduced EVA71 replication [52]. Remarkably, downregulated miR-197 during EVA71 infection increased the protein coding gene RAN, sustaining the expression of host proteins that facilitate viral replication [53]. In addition, the protein-coding gene of eukaryotic translation initiation factor 4E (eIF4E) could be repressed by EVA71-induced miR-141, leading to continuous virus replication [54]. Upregulated miR-545 promoted EVA71 replication at least partly via targeting phosphatase and tensin homolog (PTEN) and tumor necrosis factor receptor-associated factor (TRAF)6 [55]. Several miRNAs such as miR-526a [56], miRNA-548 [57], miR-146a [58], and miR-21 [59] indirectly affected EV replication by influencing host innate immunity through various independent mechanisms.

**Figure 1:**
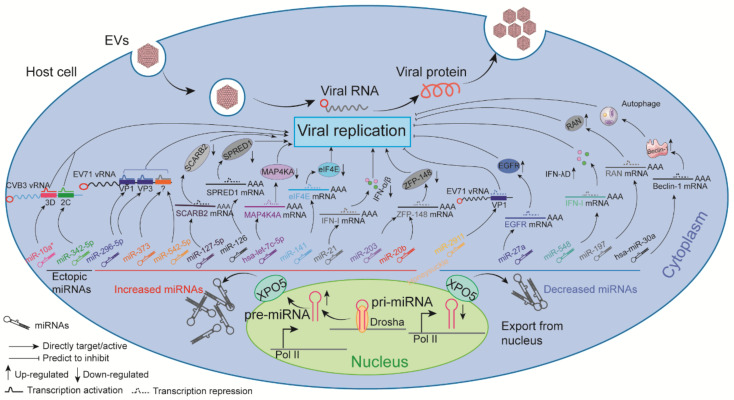
The impact of ncRNAs on EV replication. Dysregulated ncRNAs directly target the viral genome or indirectly bind to type I interferon (IFN) genes, protein-coding genes, functional molecules, and corresponding receptors to affect virus replication.

Collectively, artificial or host-coding miRNAs regulated by EVs show significant impacts on the life cycle of viruses, and these findings provide for potentially novel therapeutic tools for EV-associated diseases (Figure 1).

## 7. The Roles of ncRNAs in EV-Induced Apoptosis

**Figure 2:**
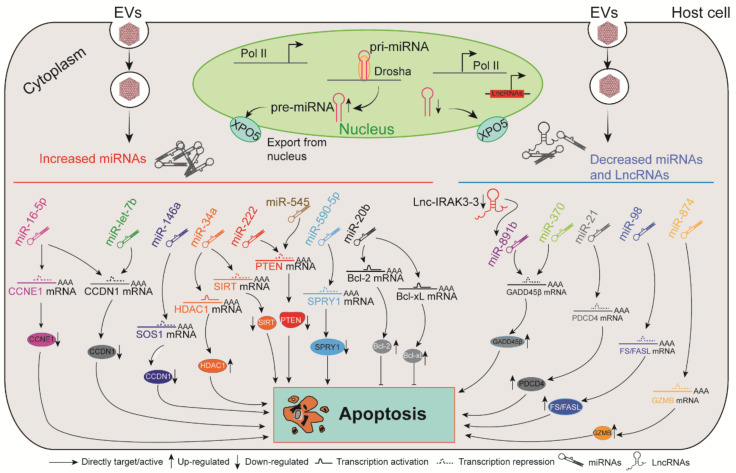
The critical roles of ncRNAs in EV-induced apoptosis. After EV invasion, dysregulated ncRNAs induced by viral infection affect the host apoptotic process by targeting apoptosis-related genes.

Apoptosis of virus-infected host cells is considered to be a pivotal mechanism against viral spread, while many viruses including EVs have consequently evolved intricate molecular strategies to overthrow these important apoptotic defenses (see Figure 2) [60], examples of which include the following and again primarily involve the activity of miRNAs.

Downregulated miR-874 facilitated EVA71-induced apoptosis in a granzyme B (GZMB)-dependent manner within Jurkat T cells, while the overexpression of miR-874 suppressed this process [61]. Son of sevenless homolog 1 (SOS1) protein is a ubiquitously expressed adapter with anti-apoptotic functions [62], and GADD45β targeting of caspase-3 and poly ADP-ribose polymerase (PARP) promotes cell apoptosis [63]. As such, miR-146a and miR-370 together coordinated EVA71 induced apoptosis through the degradation of SOS1 and GADD45β, respectively [64]. Alternatively, EVA71 reduced lnc-IRAK3-3, which can capture miR-891b to increase GADD45β-facilitated apoptosis [65]. MiR-16-5p, on the other hand, was demonstrated to be a positive feedback regulator in EVA71-induced apoptosis and a negative regulator of viral replication [66]. EVA71 also modulated the host cell cycle and proliferation by inducing endogenous miR-let-7b, directly targeting the 3′-UTR of CCND1 (cyclin D1) [67].

Apoptosis of cardiomyocytes is involved in the pathogenesis of viral myocarditis. Abundant miR-34a mitigated CVB3-induced apoptosis of cardiomyocytes by targeting silent mating type information regulation 2 homolog 1 (SIRT1)-mediated p53 pathway activity [68]. Programmed cell death 4 (PDCD4)-mediated apoptosis is an important component that propagates CVB3-induced myocarditis, and CVB3 reduced the amount of miR-21 that could directly inhibit PDCD4 in a mouse model, leading to continuous apoptosis and worsened myocarditis [69]. Intriguingly, CVB3-induced miR-20b repressed the expression of ZFP-148, which is known to regulate viral replication, while aberrant mir-20b expression induced increases in the anti-apoptosis proteins B cell lymphoma-2 (Bcl-2) and B-Cell lymphoma-extra large (Bcl-xl) [43]. Thus, CVB3 appears to induce miRNAs that balance cardiomyocyte survival vs. apoptosis to promote an environment optimal for viral replication.

Intriguingly, the extracellular release of miR-590-5p induced by CVB infection resulted in prolonged virus replication by inhibiting pro-apoptotic factors [70]. Suggesting a similar mechanism of action, circulating miR-98 was reportedly downregulated in myocarditis patients, and mir-98 can modulate cell apoptosis by interacting with the FAS/FASL gene pair [71]. PTEN is known as an apoptosis-associated protein [72]. Adenosine deaminases acting on RNA (ADAR) 1p150 plays a critical role in complexing with Dicer and increasing the amount of miRNA-222, the latter of which inhibited the expression of target gene PTEN during CVB3-induced viral myocarditis [73]. Taken together, ncRNAs play an important role in the regulation of cell fate during EV infection.

## 8. Dysregulated ncRNAs Modulate Multiple Signaling Pathways in Response to EV Infection

Available data present correlations between ncRNAs and key host pathways dysregulated during EV infection [74].

Immune responses and inflammatory cytokine production, leukocyte recruitment, or apoptosis are regulated by the nuclear factor kappa-B (NF-κB) family of transcription factors [75,76]. Bacterial components (lipopolysaccharide, LPS) activate NF-κB through the myeloid differentiation factor 88 (MyD88)-dependent pathway, leading to the upregulation of miR-146 [77]. It has been reported that CVB3-induced miR-146a regulates the NF-κB signaling pathway through the downregulation of Toll-like receptor (TLR) 3 and TRAF6, dampening antiviral immune responses [78]. Likewise, both miR-214 and miR-10 could target ITCH, an NF-κB signaling suppressor, and they further regulated the inflammatory reactions following CV infection [79,80]. Hinting at complex regulatory processes, the expression of lncRNA HIF1A-AS1 was significantly enhanced in CVB3-induced myocardium and cardiomyocytes, which promoted NF-κB signaling and subsequently induced cardiomyocyte apoptosis as well as inflammatory reactions via binding to miR-138 [81]. In addition, miR-217, miR-543, miR-9-5p, miR-93, and miR-526a have been observed to directly or indirectly affect EV pathogenesis through modulation of the NF-κB signaling pathway [56,82,83,84].

Phosphatidylinositide 3-kinase (PI3K)/Akt is an important signaling pathway controlling cell division, autophagy, survival, and differentiation [85]. Remarkably, the amount of miR-27a was significantly decreased following EVA71 infection, promoting viral replication by maintaining EGFR, and the overexpression of miR-27a could inhibit EGFR expression and the phosphorylation of Akt and Extracellular signal regulated kinase (ERK), which normally inhibit EVA71 replication [50]. In addition, EVA71-induced miR-494-3p could activate PI3K/Akt signaling by targeting PTEN [86]. As with PI3K/Akt, the mitogen-activated protein kinase (MAPK) signaling pathway is ubiquitous and regulates highly evolutionarily conserved mechanisms of eukaryotic cell function, including miRNA generation [87]. For example, the phosphorylation of transactivation response element RNA-binding protein(TRBP), a component of the miRNA complex, is mediated by the MAPK/Erk pathway [88]. In one study, MAP4K4 as an important potential candidate target was suppressed by hsa-let-7c-5p, which led to a reciprocal activation of c-Jun N-terminal kinase (JNK), which in turn facilitated the replication of EVA71 [51]. In another study, an abundance of miRNA-21 inhibited CVB3 progeny release by targeting the MAP2K3/p38 MAPK signaling pathway [89].

Additional key pathways also play a role in EV pathogenesis. The role of the Janus kinase (JAK)–signal transducer and activator of transcription (STAT) signaling pathway in normal and abnormal cellular functions has also been well studied. MiR-124 was reported to affect EV replication through modulation of the JAK–STAT signaling pathway [90]. In addition, when upregulated, miR-126 mediated cross-talk between the ERK1/2 and Wnt/β-catenin pathways, which in turn promoted CV replication [44]. MiR-217 and miR-543 were upregulated in the peripheral blood of CVB3 infections with viral myocarditis, and these endogenous miRNAs could affect myocardial injury by regulating the SIRT1/ Adenosine Monophosphate (AMP)-activated protein kinase (AMPK)/NF-κB signaling pathway [82]. EVA71 infection could also upregulate Karyopherin α2 (KPNA2), which is related to JNK1/JNK2 and p38 MAPK, by inhibiting miR-302 cluster expression [91]. In addition, upregulated miR-21 contributed to the pathogenesis of chronic viral myocarditis by regulating the transforming growth factor (TGF)–β1/Smad7 signaling pathway in mice [92].

Together, dysregulated ncRNAs induced by EVs can affect multiple signaling pathways involved with inflammation, virus replication, and host defense, as summarized in Table 3. The modulation of these signaling pathways may be beneficial for the virus and may promote viral replication, virus escape, or virus-associated disease. However, in other instances, these processes can be unfavorable to the virus, leading to an effective immune response to clear a viral infection.

## 9. The Impact of ncRNAs on Host Innate Immune Responses Induced by EVs

The condition of the host immune response to a virus determines the outcome of infection [93]. Innate immunity serves as the first line of defense against foreign dangerous substances and some pathogens [93]. In contrast to adaptive immunity, which relies on the expansion of antigen-specific lymphocytes, the innate immune system depends on a limited number of functional proteins and innate receptors [94]. The recognition of viruses is primarily initiated by a set of germline-encoded molecules referred to as pattern recognition receptors (PRRs). In turn, PRR signaling cascades immediately responds to pathogenic invasion, and in some cases, it also serves as triggers that subsequently initiate adaptive immune responses for the clearance of invading viruses [95,96]. These types of PRRs, including transmembrane Toll-like receptors (TLRs) and retinoic acid inducible-gene I (RIG-I), NOD-Like Receptors (NLRs), along with their respective signaling cascades are activated upon recognizing ‘‘microbial non-self’’ conserved molecular structures (e.g., peptidoglycan, lipopolysaccharide, viral single-stranded RNA (ssRNA) and double-stranded RNA (dsRNA), in addition to DNA) termed pathogen-associated molecular patterns (PAMPs) and dangerous immunologic molecules (e.g., unmethylated CpG DNA or pathogen-derived DNA) termed damage-associated molecular patterns (DAMPs) [14,97].

The miR-548 family was suppressed during EVA71 infection, which was identified to target the 3’UTR of IFN-λ1. In addition, miR-548 mimics transfected into cells significantly inhibited the production of IFN-λ1 induced by EVA71, pinpointing an important innate response mechanism to viral infection [57]. TRAF proteins are essential components of signaling pathways activated by TLRs [98]. Upregulated miR-628-5p upon EVA71 infection could target the 3’UTR of TRAF3, which further suppressed TRAF3-mediated IFN-β transcription in rhabdomyosarcoma cells(RD) [99]. EVA71 induced miR-545 in 293T cells and RD cells, which directly targeted the 3’UTR of TRAF6, leading to persistent viral replication [55]. It has also been reported that the expression of miR-146a, which targets interleukin-1 receptor-associated kinase 1 (IRAK 1) and TRAF6 involved in TLR signaling and type I IFN production, was significantly upregulated in EVA71-infected cells [58]. Likewise, the abundance of miR-146a facilitated viral pathogenesis by suppressing IFN-β production [58]. LncRNA MEG3 could bind to miR-223 with the target of TRAF6 to decrease M2 macrophage polarization in viral myocarditis induced by CVB3 [100].

Signaling by most TLRs is mediated by the adaptor molecule MyD88, which could thus serve as a key target by some viruses to suppress innate immunity [101]. Upregulated miR-21 induced by EVA71 inhibited the production of type I IFN by targeting MyD88 and IRAK1 [59]. Likewise, miR-30a packaging by exosomes targeted MyD88 and inhibited type I IFN production by promoting EVA71 replication in human oral epithelial cells [102]. In addition, upregulated miR-526a positively regulates RIG-I-dependent type I IFN production, and increased miR-155-5p negatively regulated the forkhead box protein O3 (FOXO3)/IRF7 axis to affect type I IFN production during EVA71 infection [56,103]. The overexpression of miR-9-5p suppressed RIG-I-mediated NF-κB signaling in several EVA71-infected cell lines [83]. The amount of miR-15 expression was significantly upregulated in H9c2 cells following CVB3 infection. The inhibition of MiR-15, which directly targets NOD-like receptor X1 (NLRX1), can reduce myocardial injury by regulating the NLRP3 inflammasome [104]. The MiR-302 cluster could reduce EVA71-induced innate immune responses by targeting the 3′UTR of KPNA2 that mediates the nuclear translocation of JNK, p38 MAPK, and NF-κB-p65, and thus, it indirectly affects these keys signaling pathways [91]. The relationship between circRNAs and immune responses in some viral infections has gradually been demonstrated [105,106]. Nonetheless, the role of circRNAs in modulating innate immune responses induced by EVs has not yet been reported.

**Figure 3:**
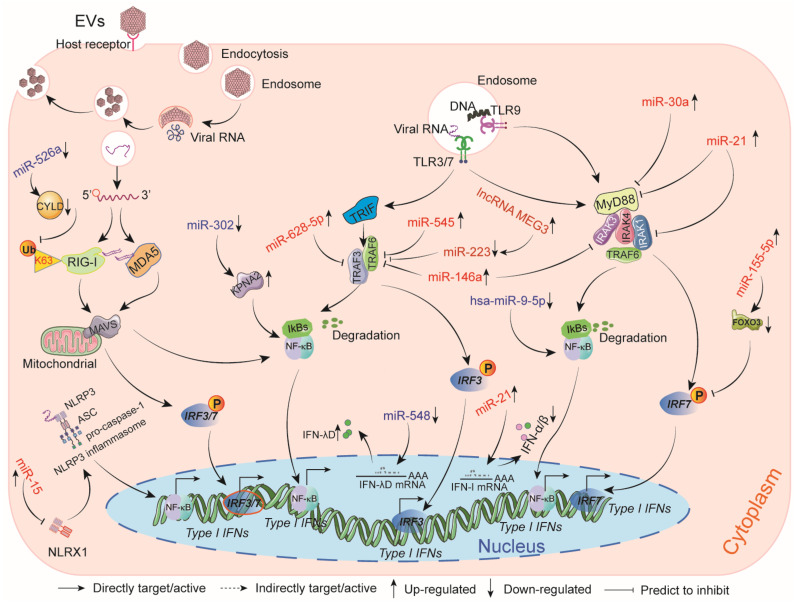
The impact of ncRNAs on host innate immune responses induced by EVs. Dysregulated ncRNAs suppress or promote host innate immune responses by either directly targeting type I type I interferon (IFN) genes or by indirectly targeting other molecules that influence these innate signals.

In summary, ncRNAs can affect EV-induced innate immune responses either by directly targeting type I IFN genes and innate signals, or by indirectly interacting with other regulators that in turn influence innate immune signals (Figure 3).

## 10. Modulation of Immune Cell Function by ncRNAs during EV Infection

Viral infection is restricted by the coordinated activation of tissue-resident and circulating immune cells [107], and EVs primarily influence cellular rather than humoral immunity to affect viral pathogenesis and clinical outcome [108].

Bioinformatics analysis of lncRNAs in leukocyte samples from acute fulminant myocarditis patients indicated that dysregulated miRNAs and lncRNAs might participate in the process of T cell activation, including T-helper 17 (Th17) cell differentiation [109]. Both miR-21 and miR-146b were upregulated in a murine model of viral myocarditis induced by CVB3, and they were demonstrated to modulate Th17 differentiation [34]. CD4 T cells play crucial roles in mediating adaptive immunity, and dysregulated CD4 T cells and miRNAs affected the pathogenesis of viral myocarditis [110]. MiR-155 deficiency reduced T cell activation and CD4 T cell proliferation in mice upon CVB3 infection [111]. Macrophages are critical contributors of innate immune responses against viral infection [112]. Increasing evidence indicates that macrophage polarization plays a critical role in the pathogenesis of CVB3-induced viral myocarditis [113,114]. A previous study reported that miR-223 expression was significantly decreased in heart-infiltrating macrophages of CVB3-infected mice, and remarkably, the overexpression of miR-223 protected these mice from myocardial injury, reduced the expression of M1 markers (iNOS, TNF-α, and CD86), and increased the expression of M2 markers (arginase-1, found in inflammatory zone 1, and CD206) in vivo and in vitro by targeting Pknox1 [114]. The lncRNA AK085865 promotes M2 macrophage polarization by acting as a negative regulator in the ILF2-ILF3 complex-mediated pri-miR-192 processing, leading to an increase in mature miR-192 production and finally attenuating susceptibility to CVB3-induced viral myocarditis [113].

Together, dysregulated miRNAs or lncRNAs can affect viral pathogenesis through regulating the immune function of T cells and macrophages during EV infection.

## 11. Circulating ncRNAs Provide Diagnostic Tools for EV-Associated Diseases

NcRNAs have been extensively studied as an emerging source of biomarkers, and the fact that their abundance in body fluids can be readily assessed makes them suitable for this purpose [115]. Most miRNAs surrounded by exosomes are more stable than mRNAs in serum, and the total amount of miRNAs remains unchanged under low temperature for a period of time [115]. Moreover, lncRNAs and circRNAs are also comparatively stable in the blood circulation due to their relatively special structures [116,117]. Accordingly, accumulating evidence indicates that circulating ncRNAs are potential candidate biomarkers for the diagnosis of EV-associated diseases.

Compared to mild cases, the expression of circulating miR-876-5p increased by 9.5-fold in severe infections with EVA71, indicating that miR-876-5p might be a prognostic biomarker for EVA71 infection [118]. The expression of circulating let-7f, miR-197, miR-223, miR-93, and miR-379 could likewise differentiate between patients with a virus (e.g., EVs) and inflammation-induced myocardial diseases and healthy donors with a specificity of over 93% [119]. In addition, cardiac miRNA profiling has been used to predict the progression of CVB3-induced viral myocarditis by assessing the risk of virus persistence and progressive clinical deterioration [120]. Higher levels of circulating miR-208b detected in the acute phase of viral myocarditis indicated that miRNA could be used as a biomarker of disease progression [121].

Together, ncRNAs have shown potential to transform the landscape of laboratory diagnosis of EVs-associated diseases.

## 12. Current Status of ncRNA-Based Therapeutic Agents for EV Infection

Certain ncRNAs bind and antagonize cellular proteins involved in antiviral responses, making their study and subsequent development of inhibitors of these ncRNAs extremely suitable for antiviral drug development. In terms of miRNAs, the overexpression of miRNAs with mimics, supplementing exogenous miRNAs, corresponding inhibitors, or modifying the binding sites of virus and miRNAs could provide potent therapeutic tools for the precise design of a new class of antiviral drugs.

As examples of the above concepts, both miR-1 and miR-133 mimics reduced cardiomyocyte apoptosis in an animal model of CVB3-induced viral myocarditis [122]. Moreover, an miR-15 inhibitor alleviated CVB3-induced apoptosis in H9c2 cells [104]. Currently, Bouchie has introduced a synthetic miR-34 mimic, the first miRNA mimic entering the Phase 1 study, to restore the suppressor function of endogenous miRNA [123]. Miravirsen, a locked nucleic acid-modified DNA phosphorothioate antisense oligonucleotide, acts on miR-122 to inhibit its function and has an antiviral effect, and the safety and activity of the drug in a Phase 1 study have been verified [124]. CVA21 is a member of pathogenic EVs with potent oncolytic potential, and yet, it causes respiratory disorders and myositis in human infections. Elizabeth et al. inserted two copies of miR-133T and miR-206T into the 3’UTR of CVA21 (the recombined virus was named miRNA-targeted CVA21), respectively, and the results showed that miRNA-targeted CVA21 retained oncolytic efficacy in vivo and exhibited a protection phenotype against fatal myositis [125]. This study provides a new paradigm for controlling the tropism of replicating viruses for therapy and offers a new modality for attenuating viruses for safer vaccines. Likewise, Barnes et al. inserted the target sequences of miR-133 and miR-206 into the coding region of the CVB3 genome between structural and nonstructural genes. This operation controlled CVB3 tissue tropism and pathogenesis in skeletal muscle and cardiomyocytes [126]. The miRNAs derived from some traditional Chinese medicines have also shown antiviral effects. For example, miR-2911 derived from *honeysuckle* soup strongly inhibited EVA71 replication by targeting the coding region of VP1 [48]. In addition, upregulated miR-1 repressed the expression of Connexin 43 (Cx43), a major gap-junction protein, in mice with viral myocarditis induced by CVB3 [127]. *Astragalus* Root dry extract (ARDE) restored Cx43 expression by targeting miR-1 in vitro and in vivo [128].

Although the above evidence implies that ncRNAs may provide novel therapeutic targets against EV infection, developing drugs to target ncRNAs will involve equally novel challenges. These include technical challenges, efficiency, safety, and clinical cost issues, which must be addressed before full clinical application of these potentially valuable therapies.

## 13. Conclusions and Perspectives

In recent years, reported outbreaks of neurological, upper or lower respiratory tract, and myocardial diseases associated with EVs have increased in frequency and size. Accumulating evidence has linked the dyregulation of ncRNAs to almost all pathophysiological processes of human diseases, including infectious diseases, and current signs of progress have yielded significant and fascinating new knowledge of them. In this review, we discussed current advances regarding the roles of ncRNAs in EV infection and the potential diagnostic and therapeutic applications of these molecules or their mimics. We described how host cell apoptosis, signaling pathways, and immune dysfunction are affected by EVs, and the novel and diverse mechanisms by which ncRNAs serve as important regulators in the EV life cycle. NcRNAs also serve as potential candidate diagnostic and therapeutic tools for EV-associated diseases. It is important to note that the effect of EVs on host ncRNAs depends on viral factors, the duration of the infection, and the cell line or tissue type. Most relevant studies have focused on the interactions between miRNAs and EVs, and current knowledge of lncRNAs and cirRNAs in EV infection is extremely limited. In addition, only a small number of studies have conducted in-depth research about carrier exosomes and the epigenetic mechanisms they influence. To better understand the cellular and molecular mechanisms involved in EV infection, a more comprehensive elucidation of the role of ncRNAs in disease pathophysiology of EVs is necessary.

Despite the comparatively short period of time since the discovery of ncRNAs, there have been enormous developments in both basic and clinical applications. It is likely that this emerging area will rapidly gain pace and develop into a new field of more specific diagnostic and therapeutic approaches to improve the level of human health care. Based on the knowledge of ncRNAs in EV infection, many barriers remain to be addressed before the use of ncRNAs in basic and clinical applications becomes practical. First, as novel diagnostic tools, a comprehensive multi-center evaluation of ncRNA sensitivity, specificity, and clinical development cost is needed. Second, specific ncRNAs may inevitably activate or repress unwanted targets of the host cell in some instances. The pharmacokinetics and pharmacodynamics of candidate ncRNA drugs will need to be tested, and toxicology evaluation is also necessary. Finally, foreign ncRNAs or modified viruses that entering human body may induce unpredictable immune responses. Addressing all barriers will help advance the clinical applications of ncRNAs in EV-associated diseases.

## Figures and Tables

**Table 1:** Classification of enteroviruses (EVs) and their corresponding diseases.

Subtype	EVs	Common Diseases Caused by EVs
Types-A	CVA6, CVA16, EVA71, other serotypes	HFMD, Herpangina, Aseptic meningitis, Pulmonary edema, Neonatal sepsis, Acute febrile disease
Types-B	CVB1-B6, 7 of the original echoviruses	HFMD, Aseptic meningitis, Respiratory disorders, Meningitis, Viral myocarditis, Herpangina, Neonatal sepsis
Types-C	polioviruses and other serotypes	Poliomyelitis, Respiratory disorders, Muscle inflammation, Neonatal sepsis
Types-D	EV-D68, EV-D70, EV-D94, EV-D111, and EV-D120	Respiratory disorders, Acute flaccid myelitis (AFM), Aseptic meningitis

**Table 2:** The aberrant expression profile of non-coding RNAs (ncRNAs) in host cells induced by EVs.

Virus Type	Model	ncRNA	Total	Up	Down	qRT-PCR	References
EVA71	in vitro	lncRNA	23	18	5	YES	[22]
EVA71	in vivo	lncRNA	104	72	32	YES	[22]
EVA71	in vitro	lncRNA	4866	2990	1876	YES	[23]
EVA71	in vitro	miRNA	4270	1718	2552	NO	[23]
EVA71	in vivo	miRNA	20	4	16	YES	[24]
EVA71/CVA16	human	miRNA	128	102	26	YES	[25]
EVA71	ex vivo	lncRNA	8541	-	-	YES	[26]
CVA16	in vitro	miRNA	1954	1825	129	YES	[27]
EVA71/CVA16	in vitro	miRNA	39/92	14/36	25/56	YES	[28]
EVA71/CVA16	ex vivo	miRNA	76/42	10/12	66/30	YES	[29]
CVB3	in vitro	lncRNA	700	431	269	NO	[30]
CVB3	in vitro	miRNA	597	-	-	YES	[31]
CVB3	in vivo	miRNA	96	33	63	YES	[32]
CVB3	in vivo	miRNA	5	4	1	YES	[33]
CVB3	in vivo	miRNA	3	2	1	YES	[34]
CVB3	human	miRNA	107	-	-	YES	[35]
CVB5	in vivo	miRNA	33	-	-	YES	[36]
CVB4	in vitro	miRNA	7	-	-	YES	[37]

Quantitative real-time polymerase chain reaction (qRT-PCR).

**Table 3:** The impact of ncRNAs on host signaling pathways following enterovirus infection.

Signaling Pathway	Influence	NcRNAs	Expression	Viral Replication	Model	References
NF-κB	inhibition	miR-146a	Upregulated	Unknown	in vitro	[78]
	inhibition	miR-217	Upregulated	Unknown	in vivo; in vitro	[82]
	inhibition	miR-543	Upregulated	Unknown	in vivo; in vitro	[82]
	inhibition	miR-9-5p	Downregulated	Unknown	in vivo; in vitro	[83]
	activation	miR-214	Upregulated	Unknown	in vivo;	[79]
	activation	LncRNA HIF1A-AS1	Upregulated	Unknown	in vivo; in vitro	[81]
	unknown	miR-93	Upregulated	Unknown	in vivo; in vitro	[84]
	activation	miR-526a	Upregulated	Inhibition	in vitro	[56]
PI3K/AKT	inhibition	miR-27a	Downregulated	Inhibition	in vitro	[50]
	activation	miR-494-3p	Upregulated	Promotion	in vitro	[86]
MAPK	inhibition	miR-21	Upregulated	Unknown	in vivo; in vitro	[89]
	inhibition	let-7c-5p	Upregulated	Promotion	in vitro	[51]
JAK/STAT	inhibition	miR-124	Upregulated	Promotion	in vitro	[90]
	inhibition	miR-217	Upregulated	Unknown	in vivo; in vitro	[82]
	inhibition	miR-543	Upregulated	Unknown	in vivo; in vitro	[82]
Wnt/β-catenin	inhibition	miR-126	Upregulated	Promotion	in vitro	[44]
AMPK	inhibition	miR-217	Upregulated	Unknown	in vivo; in vitro	[82]
	inhibition	miR-543	Upregulated	Unknown	in vivo; in vitro	[82]
JNK	inhibition	miR-302	Downregulated	Inhibition	in vivo; in vitro	[91]
	activation	let-7c-5p	Upregulated	Promotion	in vitro	[51]
TGF-β1/Smad7	inhibition	miR-21	Unknown	Unknown	in vivo;	[92]

## Data Availability

Not applicable.
